# Green Tea Attenuates Oxidative Stress and Downregulates the Expression of Angiotensin II AT_1_ Receptor in Renal and Hepatic Tissues of Streptozotocin-Induced Diabetic Rats

**DOI:** 10.1155/2012/409047

**Published:** 2012-11-14

**Authors:** Martha Thomson, Khaled Al-Qattan, Mohamed H. Mansour, Muslim Ali

**Affiliations:** Department of Biological Sciences, Faculty of Science, Kuwait University, P.O. Box 5969, Safat 13060, Kuwait

## Abstract

This study investigates the potential of green tea to modulate oxidative stress and angiotensin II AT_1_ receptor expression in renal and hepatic tissues of diabetic rats. Three groups of rats were studied after 8 weeks following diabetes induction: normal, streptozotocin-induced diabetic (diabetic control), and green-tea-treated diabetic rats. Total antioxidant, catalase, and malondialdehyde levels were assayed by standard procedures. Levels of AT_1_ receptor labeling, in renal and hepatic tissues of the three rat groups, were immunohistochemically investigated using an anti-AT_1_ receptor antibody. Levels of total antioxidant and catalase were significantly reduced, whereas malondialdehyde levels and AT_1_ receptor labeling were significantly increased in renal and hepatic tissues of diabetic control rats compared to normal rats. Compared to diabetic control rats, total antioxidant and catalase levels were significantly increased, whereas malondialdehyde levels and AT_1_ receptor labeling in the green-tea-treated diabetic group were significantly reduced throughout hepatic lobules and renal cortical and medullary vascular and tubular segments to levels comparable to those observed in normal rats. The capacity of green tea to modulate diabetes-induced oxidative stress and AT_1_ receptor upregulation may be beneficial in opposing the deleterious effects of excessive angiotensin II signaling, manifested by progressive renal and hepatic tissue damage.

## 1. Introduction

Brewing dried leaves and buds of the plant *Camellia sinensis* produces tea; one of the most commonly consumed beverages worldwide [1]. Green tea, about 20% of the tea produced annually, is predominantly consumed in Asian countries but has become increasingly popular in Western countries during the last decade [2]. All types of tea beverages contain polyphenols specifically flavonoids. Green tea contains mainly catechins, members of the flavan-3-ol class of flavonoids. The four major catechins in green tea are (−)-epicatechin (EC), (−)-epicatechin gallate (ECG), (−)-epigallocatechin (EGC), and (−)-epigallocatechin gallate (EGCG). A typical green tea serving contains about 90–100 mg of catechins of which EGCG accounts for 50–80% [3].

Many medicinal properties have been ascribed to green tea including anticancer, antidiabetic, and antihypertensive effects [4, 5]. Many of these beneficial effects have been attributed to the presence of catechins, which are potent antioxidants. Since oxidative stress has also been strongly correlated to the increased incidence of diseases such as cancer, cardiovascular diseases, and even in the aging process, green tea has been studied as a potential ameliorative agent in these conditions. Several studies have demonstrated that green tea components scavenge reactive oxygen species formed *in vitro* [6]. In addition, animal studies have suggested that green tea might protect against the development of coronary heart disease by reducing blood glucose levels and body weight [7]. Catechins also reduced plasma triglyceride levels in an oral glucose tolerance test in normal rats [5]. Several human and animal studies suggested that green tea and its flavonoids have antidiabetic effects [8, 9]. 

Diabetes mellitus is the most common metabolic disorder that causes nearly 7% of all worldwide deaths annually [10]. Chronic hyperglycemia in diabetes is associated with long-term damage, dysfunction, and eventually the failure of organs, especially eyes, kidneys, nerves, and the cardiovascular system [11–13]. Dyslipidemia is also involved in the development of cardiovascular complications in diabetes, which are the major causes of morbidity and mortality [14, 15]. Experimental and clinical studies have shown that diabetic hyperglycemia is a major source of oxidative stress, and that increased generation of free radicals plays a major role in the pathogenesis and complications of diabetes [16–19]. Glucose autoxidation, formation of advanced glycation end products, and increased activity of the sorbitol pathway have been suggested as the basic mechanism by which chronic hyperglycemia promotes increased oxidative stress [18]. Oxidative stress in diabetes leads to tissue damage, with lipid peroxidation, inactivation of proteins, and protein glycation as intermediate mechanisms for its complications. Damage to small blood vessels (microvascular disease) can lead to the development of diabetic nephropathy which is characterized by progressive loss of kidney function, as a result of reduction in glomerular filtering capacity leading to proteinuria and albuminuria. Between 25 and 40% of diabetic patients have nephropathy, and 30–40% of newly diagnosed cases of end-stage renal disease requiring dialysis are characterized as having increased oxidative stress [20, 21] attributed to diabetes [22, 23]. As a prevalent and serious complication of diabetes, prevention or reversal of diabetic nephropathy would improve the prognosis of diabetic patients.

As manifested in diabetic patients and animal models, the renin-angiotensin system has been shown to progressively augment proteinuria and accelerate decline in renal function [24]. Through its multiple interactions with its specific AT_1_ receptor, angiotensin II (Ang II), the major active product of this system synthesized both systemically and within the kidney, is a mediator of progressive injury in diabetic nephropathy. As previously documented, the hyperglycemia-induced AT_1_ receptor upregulation in the adrenal and kidney mediates the increase of Ang II local effects in stimulating aldosterone production and over-activating renal sodium transporters causing sodium retention and hypertension [25]. In the diabetic liver, Ang II induces hepatic inflammation and stimulates a range of fibrogenic actions, including cell migration, cell proliferation, the production of intracellular reactive oxygen species, secretion of proinflammatory cytokines, and collagen synthesis predominantly through the AT_1_ receptor [26]. Within the kidney, the upregulated AT_1_ receptor signaling increases vascular resistance, glomerular capillary pressure, and mechanical stretch-induced glomerular injury and stimulates production of reactive oxygen species and extracellular matrix in the mesangium and tubulointerstitium [25, 27], which collectively perpetrates the development of diabetic nephropathy. Currently, there is accumulating evidence that pharmacological interference with the chronic activation of the renin-angiotensin system represents an important therapeutic target, which may provide incremental end-organ protection. In particular, the use of AT_1_ receptor blockers, solely or among other regimens, may prove beneficial in opposing the detrimental effects of AT_1_ receptors in the progression of diabetic nephropathy as well as other diabetic complications [28].

The present study was designed to study the beneficial effects of green tea in the streptozotocin- (STZ-) induced diabetic rat model. In particular, the current study focused on the antioxidant potential and putative modulatory effect of green tea on AT_1 _receptor expression in ameliorating diabetic complications in STZ-induced diabetic rats.

## 2. Materials and Methods 

### 2.1. Animal Model and Experimental Design

Male Sprague-Dawley rats weighing 150–180 g were used for the study. The study conformed to the National Research Council (NRC) Guide for the Care and Use of Laboratory Animal [29]. All animals were maintained on a 12 h light/dark cycle and a temperature of 22 ± 1°C. All animals received diet and water or tea ad libitum. Thirty rats were injected with streptozotocin (STZ). The remaining rats were used as normal controls. For induction of diabetes, rats were weighed, fasted overnight, and injected intraperitoneally (IP) with a single dose of 60 mg STZ/kg body weight in a constant volume of 0.5 mL citrate buffer. The rats were divided into 3 groups (*n* = 8) and were treated for 8 weeks. Group 1 was normal control rats drinking only water (normal control), group 2 was diabetic rats drinking only water (diabetic control), and group 3 was diabetic rats drinking 0.1% green tea (diabetic + green tea). A drop of blood was obtained from each rat by tail-vein puncture before STZ injection and weekly after STZ injection to determine blood glucose levels. The blood glucose level was measured using a one touch UltraEasy glucometer (LifeScan, Johnson & Johnson, UK), and rats were determined to be diabetic if they had elevated plasma glucose concentrations ≥300 mg/dL five days postinjection, as described previously [30].

### 2.2. Antibodies and Preparation of Green Tea Extract

Polyclonal anti-AT_1_ receptor antibody (sc-1173, rabbit IgG specific to an epitope mapping within the N-terminal extracellular domain of the human AT_1_ polypeptide) and peroxidase-conjugated goat anti-rabbit IgG antibody were purchased from Santa Cruz Biotechnology, Inc. (Santa Cruz, CA, USA). An eight-amino-acid peptide, corresponding to amino acids 14–21 (Ile-Gln-Asp-Asp-Cys-Pro-Lys-Ala) of the first extracellular domain, as deduced from the published AT_1_ receptor cDNA sequence [31], was synthesized manually on the base-labile linker 4-(hydroxymethyl)-benzoyloxymethyl and supplied by The Protein/DNA Technology Center (The Rockefeller University, NY, USA). The octapeptide was conjugated to bovine serum albumin (BSA) and coupled to CNBr-activated Sepharose 4B as described previously [32]. 

 A 0.1% green tea extract (Al-Wazah Tea, Ceylon) was prepared by weighing 1 gram of green tea leaves and suspending them in 1000 mL of hot boiled distilled water. The extract was then stirred for 15 min and filtered using cheesecloth. The tea extract was prepared daily. The water content of the control group bottles was also changed daily. After 8 weeks, rats were sacrificed under sodium pentobarbitone anesthesia (10 mg/kg, Sagatal, May and Baker UK), blood was collected via cardiac puncture, and serum was obtained and stored in small aliquots at –80°C for further analysis. The liver and kidneys of each rat were individually collected, weighed, and either placed in vials containing 3 mL of Bouin's fixative for 24–48 h at room temperature or placed in separate small plastic bags and stored at −40°C for further analysis.

### 2.3. Preparation of Tissue Homogenates

One gram of the kidney or liver excised from normal, control diabetic, and/or green-tea-treated diabetic rats was cut into small pieces and mixed with 3 mL Tris HCl (0.05 M, pH = 7.6) buffer. The mixture was homogenized, allowed to stand on ice for few minutes, and then centrifuged for 15 min at 8000 ×g at 4°C. The supernatant stored in small aliquots at −40°C for further analysis.

### 2.4. Biochemical Assays

Protein concentration of serum and tissue homogenates was determined by the Coomassie blue dye-binding method using bovine serum albumin as standard [33]. Total serum or tissue antioxidant levels were determined as described in Drobiova et al. [30]. Tissue malonaldehyde (MDA) levels were determined according to the method of Ohkawa and coworkers [34]. Catalase activity was quantified by the method of Abei [35].

### 2.5. Preparation of Tissue Sections

Fixed kidneys and Livers of normal, control diabetic, and/or green-tea-treated diabetic rats were individually carried through a routine paraffin embedding technique, which included dehydration through a series of ethanol concentrations 50%, 70%, 90%, and 100%, clearing in toluene, embedding in paraffin wax, and finally sectioning of the paraffin blocks into 3-4 *μ*m thick sections on a rotary microtome. The sections were picked up on clean slides after spreading them in a water bath at 40°C. The slides were air-dried to be used for subsequent staining.

### 2.6. Labeling of Tissue Sections with Anti-AT_1_ Receptor Antibody

Tissue sections were examined for AT_1_ receptor distribution by an indirect immunohistochemical labeling technique. Tissue sections were dewaxed in xylene, hydrated with a series of 90%, 75%, and 60% ethanol, and washed with PBS, pH 7.2. Sections were individually labeled for 45 min in a humidified chamber with 300 *μ*L of rabbit anti-AT_1_ receptor antibody (diluted to 1 : 100 with PBS, pH 7.2). After three washes with 200 *μ*L PBS, pH 7.2, the sections were incubated for 45 min with 300 *μ*L of peroxidase-conjugated goat anti-rabbit IgG antibody (diluted to 1 : 200 in PBS, pH 7.2) then washed three times with 200 *μ*L PBS, pH 7.2, followed by a 5 min treatment with 400 *μ*L of 3,3-diaminobenzidine tablets (fast DAB) reconstituted in water. All sections were counterstained with 100 *μ*L haematoxylin (Gill number1) for 1 min and examined by light microscopy for positive labeling of cells expressing AT_1_ receptors. Control sections were identically stained by replacing the specific antibody with anti-AT_1_ antibody preabsorbed with an AT_1_ octapeptide/BSA complex-coated CNBr-activated Sepharose 4B beads. To exclude nonspecific binding, all sections were treated with 300 *μ*L of 0.3% hydrogen peroxide in PBS, pH 7.2 for 10 min to block endogenous peroxidase, followed by a 30-min block with 300 *μ*L of 5% normal goat serum in PBS, pH 7.2, prior to the application of the anti-AT_1_ antibody, to minimize off-target staining. Tissue sections prepared from normal control, diabetic control, and green-tea-treated diabetic rats were processed in parallel with strictly uniform incubation times and reagent volumes to ensure standardization of labeling between samples. Slides were quantitatively examined by using the Image-Pro Plus 5.1 software program (Media Cybernetics, Silver Spring, MD). Automatic object counting and measuring processes were used to quantify labeled areas, and values expressed as *μ*m^2^. Each slide was analyzed for AT_1_ receptor labeling in the liver and in the renal cortex and outer medulla, with three separate fields viewed in each region and four independent samples for each group. Data were expressed and analyzed as indicated below. Representative microphotographs were taken using Olympus AH-3 automated microscope (Tokyo, Japan), equipped with an Olympus Vanox camera.

### 2.7. Statistical Analysis

The data are expressed as mean ± standard error of the mean (SEM). The test of analysis of variance (ANOVA) with post hoc STD was employed to examine differences among readings between groups. The *P* < 0.05 level was selected as the level of significance. All statistical analyses were performed using SPSS (version 19). 

## 3. Results

### 3.1. Effect of Drinking Green Tea on Blood Glucose Level and Body Weight

**Figure 1:**
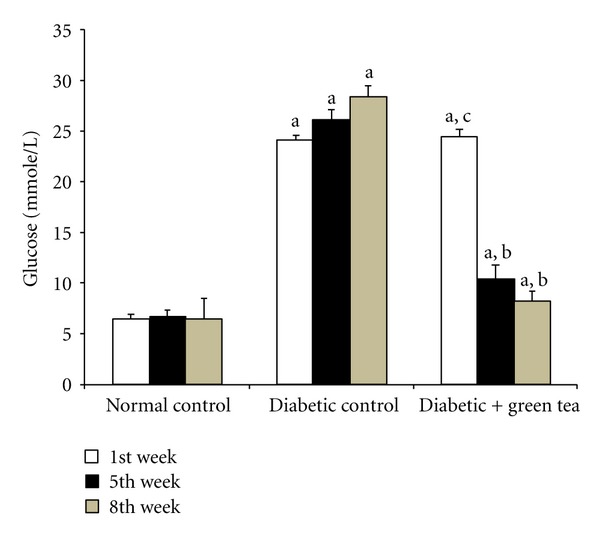
Effect of green tea on blood glucose in diabetic rats. Blood glucose levels were measured at weeks 1, 5, and 8 of the experiment in normal control rats, STZ-induced diabetic rats (diabetic control), and 0.1% green-tea-treated STZ-induced diabetic rats (diabetic + green tea). Results are expressed as means ± SEM, *n* = 8. ^a^Significantly different from normal control. ^b^Significantly different from diabetic control. ^c^No significant difference from diabetic control.

**Figure 2:**
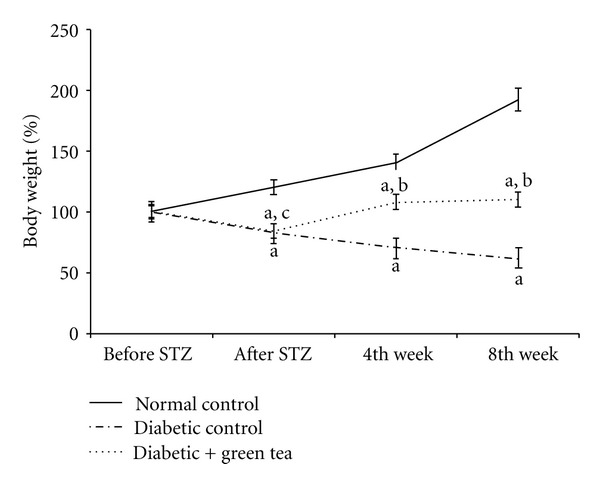
Effect of green tea on change in body weight in diabetic rats. Weights were taken for normal control rats, STZ-induced diabetic rats (diabetic control), and 0.1% green-tea-treated STZ-induced diabetic rats (diabetic + green tea). The animals were weighed before STZ injection (Before STZ), 1 week after STZ injection (after STZ), and at weeks 4 and 8 of the experiment. Weights are plotted as percentiles with the starting, weights all standardized to 100%. ^a^Significantly different from normal control. ^b^Significantly different from diabetic control. ^c^No significant difference from diabetic control.

Figure 1 shows the effect of drinking green tea extract on blood glucose level. Blood glucose levels in diabetic control rats were significantly increased compared to normal control rats during the entire experimental period. In comparison, the green tea drinking rats showed significantly decreased blood glucose compared to diabetic control rats by 5 and 8-weeks. In comparison, Figure 2 depicts body weight changes during the 8 week experiment. Figures 1 and 2 show that as blood glucose levels increased in diabetic control rats, body weight decreased compared to normal control rats that nearly doubled their weight during the 8-week experiment (Figure 2). In comparison, green-tea-treated diabetic rats have lowered blood glucose levels and increased body weight compared to the diabetic controls.

### 3.2. Effect of Drinking Green Tea on Kidney and Liver Antioxidants, MDA, and Catalase

**Figure 3:**
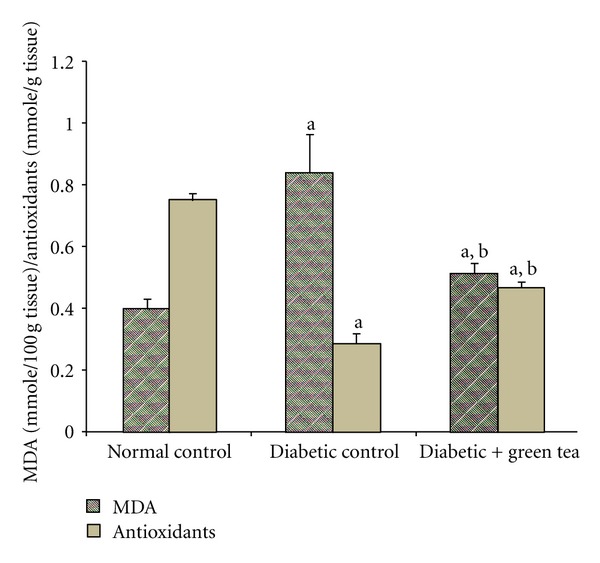
Effect of green tea on kidney MDA and antioxidants in diabetic rats. Kidney MDA and antioxidant levels were measured at the end of week 8 of the experiment in normal control rats, STZ-induced diabetic rats (diabetic control), and 0.1% green-tea-treated STZ-induced diabetic rats (diabetic + green tea). Results are expressed as means ± SEM, *n* = 8. ^a^Significantly different from normal control. ^b^Significantly different from diabetic control.

Figure 3 shows the effect of drinking green tea extract on the kidney total antioxidants and MDA levels. Kidney tissue exhibited a simultaneous significant decrease in total antioxidants and increase in MDA in diabetic control rats drinking water compared to normal control rats. The diabetic rats drinking green tea showed a significant increase in total antioxidant levels and decrease in MDA when compared to diabetic control rats.

**Figure 4:**
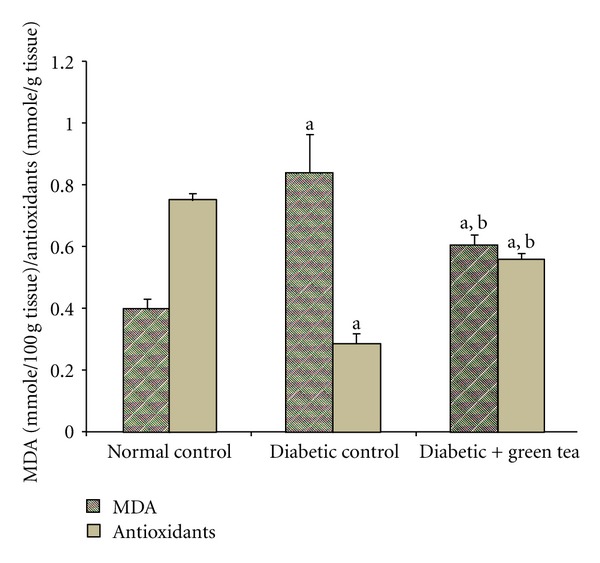
Effect of green tea on liver MDA and antioxidants in diabetic rats. Liver MDA and antioxidant levels were measured at the end of week 8 of the experiment in normal control rats, STZ-induced diabetic rats (diabetic control), and 0.1% green-tea-treated STZ-induced diabetic rats (diabetic + green tea). Results are expressed as means ± SEM, *n* = 8. ^a^Significantly different from normal control. ^b^Significantly different from diabetic control.

Liver MDA levels were significantly higher (Figure 4) as a result of STZ induction of diabetes (diabetic controls). In contrast, diabetic rats that drank green tea had liver MDA levels significantly lower than diabetic control rats. In contrast, liver total antioxidant levels were significantly decreased in diabetic control rats (Figure 4) and significantly elevated in diabetic rats drinking green tea.

Table 1 shows the effect of drinking green tea on kidney and liver catalase activity. Kidneys and livers of diabetic control rats exhibited a significant decrease in catalase activity compared to normal control rats. However, treating diabetic rats with green tea extract partially reversed this with a significant increase in both kidney and liver catalase observed in green tea drinking rats compared to diabetic control rats.

### 3.3. Effect of Drinking Green Tea on AT_1_ Receptor Expression in the Kidney and Liver

**Figure 5:**
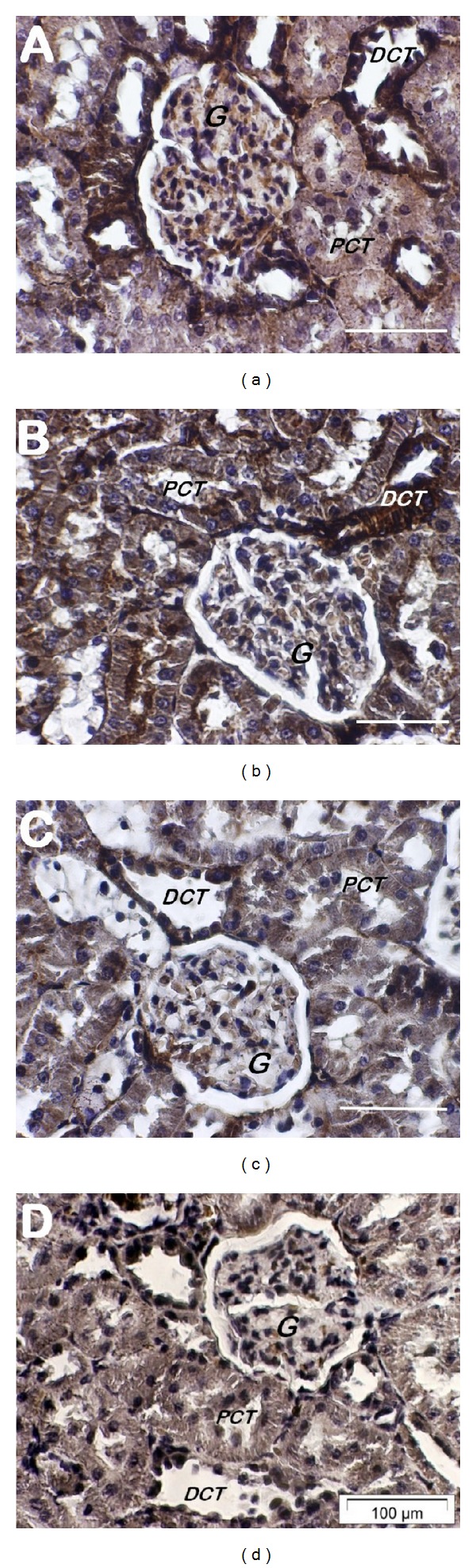
Immunohistochemical localization of AT_1_ receptor expression in the renal cortex of normal control (a), diabetic control (b), and green-tea-treated diabetic (c) rats. Renal sections were treated with the polyclonal anti-AT_1_ receptor antibody and counterstained with hematoxylin as detailed in Materials and Methods. In (a), positive labeling was detected with marked intensity in Bowman's capsule and distal convoluted tubules epithelia, but was of moderate intensity among glomeruli and uniformly diffused in proximal convoluted tubules epithelia. In (b), the labeling was maintained at a high intensity in the epithelial lining of the distal convoluted tubules, but was markedly reduced in Bowman's capsule and glomeruli as opposed to its enhanced intensity in the apical side of the proximal convoluted tubules epithelia. In (c), the labeling was almost lacking in Bowman's capsule and glomeruli and appeared as faded patches at the basolateral side of some of the proximal convoluted tubules epithelia, but was of persistent high intensity confined to the distal convoluted tubules epithelia. No specific labeling was observed in renal sections labeled with the anti-AT_1_ antibody preabsorbed with an AT_1_ octapeptide/BSA complex (d). Images are representative of four different samples for each rat group. DCT: distal convoluted tubule, G: glomerulus, and PCT: proximal convoluted tubule. Bar = 100 *μ*m, ×100.

In immunohistochemical labeling experiments of kidney sections of the three rat groups, light microscopy revealed that variable patterns of AT_1_ receptor labeling were primarily associated with the cortex as well as the inner stripe of the outer medulla. As depicted in Figure 5(a), cortical nephron segments of normal control rats exhibited AT_1_ receptor labeling of marked intensity in the outer rim of Bowman's capsule and the entire epithelial lining of the distal convoluted tubules, but of moderate intensity among glomeruli and uniformly diffused in the epithelial lining of the proximal convoluted tubules. In diabetic control rats, AT_1_ receptor labeling was maintained at a high intensity in the epithelial lining of the distal convoluted tubules, but was markedly reduced in Bowman's capsule and glomeruli as opposed to its enhanced intensity in the epithelial lining of the proximal convoluted tubules, particularly at the apical side (Figure 5(b)). On the other hand, except for the persistent high intensity of the AT_1_ receptor labeling confined to the epithelial lining of the distal convoluted tubules, the specific labeling was almost lacking in Bowman's capsule and glomeruli and appeared as faded patches at the basolateral side of some of the epithelial cells lining the proximal convoluted tubules in green-tea-treated diabetic rats (Figure 5(c)).

**Figure 6:**
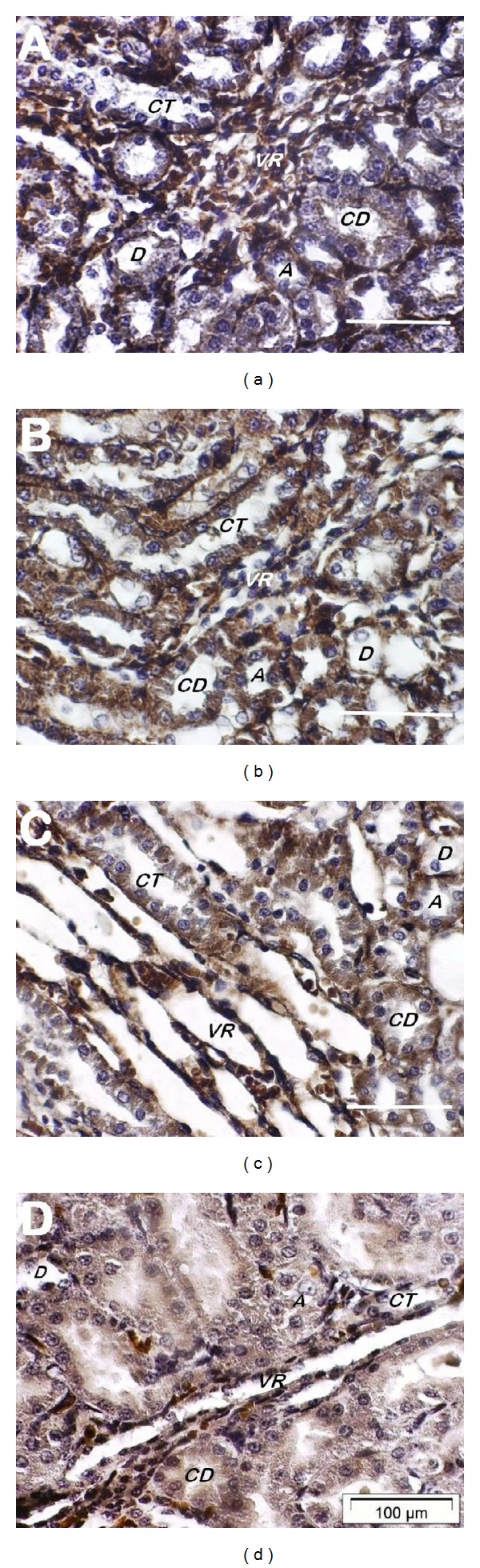
Immunohistochemical localization of AT_1_ receptor expression in the outer medulla of the kidney of normal control (a), diabetic control (b), and green-tea-treated diabetic (c) rats. Renal sections were treated with the polyclonal anti-AT_1_ receptor antibody and counterstained with hematoxylin as detailed in Materials and Methods. In (a), the labeling was evident in vasa recta bundles and, to a lesser extent, the basolateral side of collecting tubules and the apical side of collecting ducts, whereas Henle's loop segments were not labeled. In (b), the labeling was of relatively low intensity in the vasa recta bundles but was evident in the epithelial lining of collecting tubules and ducts as well as Henle's loop segments. In (c), labeling with faded intensity was observed in vasa recta bundles, and confined to the basolateral side of collecting tubules, collecting ducts, and Henle's loop epithelia. No specific labeling was observed in kidney tissue sections labeled with the anti-AT_1_ antibody preabsorbed with an AT_1_ octapeptide/BSA complex (d). Images are representative of four different samples for each rat group. A: ascending limb of Henle's loop, CD: collecting duct, CT: collecting tubule, D: descending limb of Henle's loop, and VR: vasa recta bundle. Bar = 100 *μ*m, ×100.

As in the cortical regions, distinct labeling patterns of the AT_1_ receptor were also observed in the inner stripe of the outer medulla of the three rat groups. In normal control rats (Figure 6(a)), binding of the anti-AT_1_ receptor antibody was evident in interstitial cells outlining the vasa recta bundles as well as the basolateral side of collecting tubules and, to a lesser extent, the apical side of collecting ducts, without any obvious involvement of the epithelial elements of ascending or descending Henle's loop segments. Compared to normal control rats, AT_1_ receptor labeling was of relatively lower intensity in interstitial cells outlining the vasa recta bundles in diabetic control rats, but was accentuated in the cytoplasm of the entire epithelial lining of collecting tubules, collecting ducts, and Henle's loop segments (Figure 6(b)). As shown in Figure 6(c), AT_1_ receptor labeling in interstitial cells outlining the vasa recta bundles was of significantly reduced intensity and was apparently faded and confined to the basolateral side of collecting tubules, collecting ducts, and Henle's loop segments in green-tea-treated diabetic rats. None of these specific cortical and medullary labeling patterns were observed in renal sections treated with anti-AT_1_ antibody preabsorbed with AT_1_ octapeptide/BSA complex-coated CNBr-activated Sepharose 4B beads (Figures 5(d) and 6(d)). Quantification of AT_1_ receptor staining by computer-based image analysis revealed a significant increase in expression of AT_1_ receptors in the renal cortical and outer medullary regions of the control diabetic group compared with the normal control rats. Green-tea-treated diabetic rats were marked by a return of AT_1_ receptor labeling to levels significantly lower than the control diabetic group and comparable to those observed in normal control rats (Table 2).

**Figure 7:**
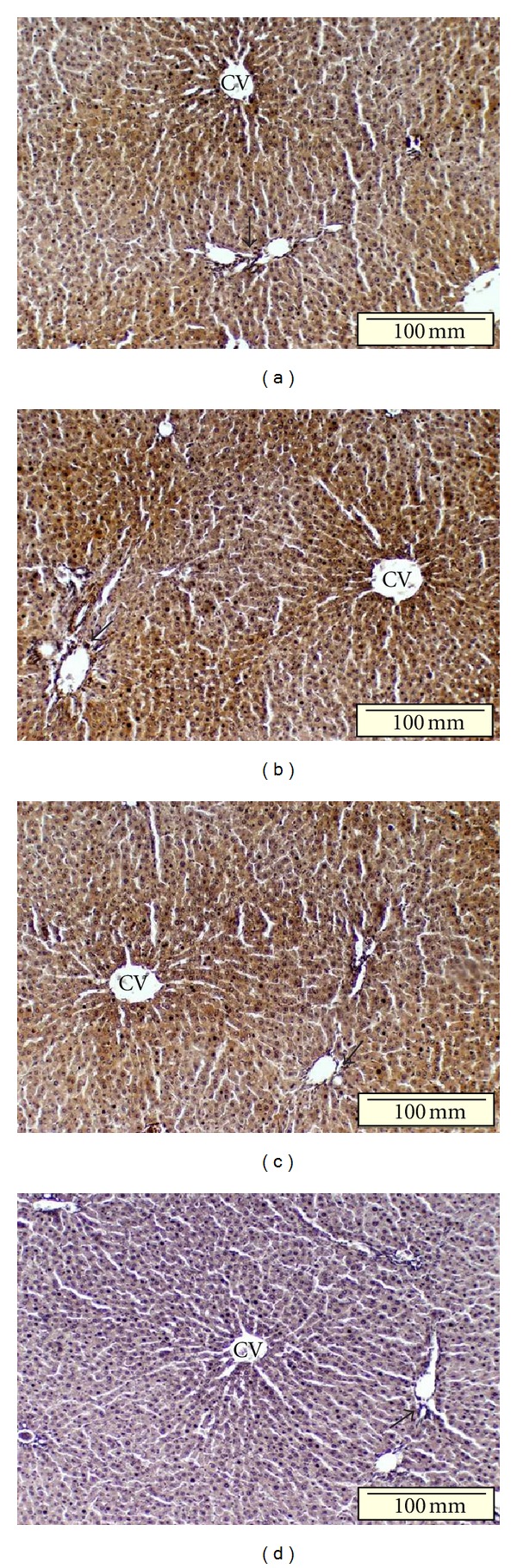
Immunohistochemical localization of AT_1_ receptor expression in the liver of normal control (a), diabetic control (b), and green-tea-treated diabetic (c) rats. Liver sections were treated with the polyclonal anti-AT_1_ receptor antibody and counterstained with hematoxylin as detailed in Materials and Methods. In both (a) and (c), moderate AT_1_ receptor labeling was selectively observed among hepatocytes arranged around the central vein (CV) within hepatic lobules. In (b), AT_1_ receptor labeling with higher intensity was observed among hepatocytes as well as bile ducts and vessels in portal tracts (arrowheads). No specific labeling was observed in liver sections labeled with the anti-AT_1_ antibody preabsorbed with an AT_1_ octapeptide/BSA complex (d). Images are representative of four different samples for each rat group. Bar = 100 *μ*m, ×100.

In liver sections, moderate AT_1_ receptor labeling was primarily observed among hepatocytes arranged around the central vein within hepatic lobules, but was less evident in the bile duct and vessels within portal tracts in normal control as well as green-tea-treated diabetic rats (Figures 7(a) and 7(c)). In contrast, a more intense AT_1_ receptor labeling, involving more hepatocytes in addition to bile ducts and vessels in portal tracts, was observed in control diabetic rats (Figure 7(b)). None of these specific labeling patterns were observed with liver sections treated with anti-AT_1_ antibody preabsorbed with AT_1_ octapeptide/BSA complex-coated CNBr-activated Sepharose 4B beads (Figure 7(d)). As depicted in Table 2, quantification of AT_1_ receptor staining by computer-based image analysis revealed a significant increase in the expression of AT_1_ receptors in the liver of control diabetic rats compared with either the normal control group or the green-tea-treated diabetic group. 

## 4. Discussion

The present study was conducted to evaluate the role of green tea as a natural agent of an ameliorative potential to oxidative stress and progressive tissue damage in STZ-induced diabetic rats. Experimental diabetes induced by STZ is a well-established method used to evaluate the mechanisms involved in the alterations of physiopathology observed in diabetic patients. STZ destroys pancreatic *β* cells, resulting in a diabetic syndrome, similar to that seen in human type1 diabetes and characterized by hyperglycemia, hypoinsulinemia, glucosuria, and loss in body weight [36–38]. In this study as in previous studies, the observed hyperglycemia in STZ-induced diabetic rats was paralleled by 40% reduction in body weight, whereas green tea extract treatments resulted in a significant reduction in blood glucose levels and prevention of weight loss in these animals. These observations are in accordance with the documented role of green tea in the regulation of body weight [38] and furthermore confirm the hypoglycemic effects of green tea reported in several other studies [7, 39, 40]. Green tea polyphenols have been shown to improve glucose tolerance in normal and obese rats and db/db mice [2, 41]. In addition, green tea has been reported to enhance basal and insulin-stimulated glucose uptake in rat adipocytes [42]. The major green tea catechin, EGCG, was shown to inhibit intestinal glucose uptake by sodium-dependent glucose transporter SGLT1 [43], whereas a catechin-rich green tea extract and EGCG have been reported to mimic insulin by decreasing the expression of genes that control gluconeogenesis [44]. Collectively, the antihyperglycemic effect of green tea observed in these studies suggests an important clinical relevance to diabetes treatment.

Increased oxidative stress can be the result of several diabetes-induced abnormalities, including autoxidation of glucose, the formation of advanced glycation end products, and impairment of the scavenging system [18]. The impaired scavenging system is always associated with reduced antioxidant capacity, abnormal activity or reduced expression of antioxidant enzymes in diabetes [19]. Among various antioxidant enzymes, catalase plays an important role in balancing the generation of reactive oxygen species and the overall tissue antioxidant capacity. In our study, the total antioxidant activity and catalase activity in the kidney and liver tissues were significantly compromised in diabetic rats compared to normal controls. This was paralleled by an increase in lipid peroxidation as evidenced by the elevated levels of MDA detected in kidney and liver tissues in diabetic rats compared to normal controls. Treatments of diabetic rats with green tea significantly increased both antioxidant levels and catalase activity, which was reflected by significantly lowering MDA levels in kidney and liver tissues compared to diabetic control rats. These observations are consistent with several studies showing the potential of green tea and/or catechins in reducing oxidative stress by either increasing total plasma antioxidant activity [45–47] or reducing MDA levels in tissues [48]. However, Hininger-Favier and coworkers [47] did not observe any change in liver MDA levels after treatment of insulin-resistant rats with green tea extract. Similarly, Yokazawa and coworkers reported no significant change in kidney MDA levels after green tea treatment of diabetic rats [49]. However, these conflicting findings might be due to differences in doses, concentrations of green tea, time of exposure, and type of green tea as well as the degree of hyperglycemia and the detection methods employed.

In diabetic patients and animal models, the altered expression of the Ang II AT_1_ receptor has been implicated as a pivotal factor in the development of early changes associated with diabetic end-organ damages, particularly nephropathy [24–27]. In the present study, AT_1_ receptor expression in the kidney and liver tissues was investigated immunohistochemically using a polyclonal anti-AT_1_ antibody of proven specificity to an epitope mapping within the N-terminal extracellular domain of the AT_1_ polypeptide [50]. In normal rats, the binding of this antibody was predominately observed among hepatocytes arranged around the central vein within hepatic lobules, in discrete kidney regions, including the glomeruli and proximal and distal convoluted tubules in the cortex, in interstitial cells outlining the vasa recta bundles, and to a lesser extent, collecting tubules and collecting ducts in the inner stripe of the outer medulla. These hepatic and renal sites corresponded well to those shown to express detectable levels of the AT_1_ receptor by *in vitro* autoradiographic localization, *in situ* hybridization, and semiquantitative reverse transcription polymerase chain reaction [51]. Confirming the documented effect of hyperglycemia on AT_1_ receptor expression [24, 27, 50], STZ treatment in the present study was paralleled by alterations in the pattern of AT_1_ receptor expression in both renal and hepatic tissues. Compared to normal rats, a significant increase in AT_1_ receptor expression was observed among the cytoplasmic and apical aspects of the proximal convoluted tubule epithelia in the renal cortex, the interstitial cells outlining the vasa recta bundles and tubular segments including Henle's loop and collecting tubules and ducts within the inner stripe of the outer renal medulla, and involved more frequent hepatocytes in addition to bile ducts and vessels in portal tracts in the liver of STZ-induced diabetic rats. As previously indicated, the upregulatory shift in AT_1_ receptor expression in the renal cortex leads to higher stimulation of Na-K-ATPase and Na/H exchanger 3 in proximal segments leading to sodium retention and subsequent hypertension [52], as well as increased mechanical stretch-induced glomerular injury culminating in the development of glomerulosclerosis and proteinuria [27]. Overstimulated Ang II signaling through upregulated AT_1_ receptors in the outer renal medulla and hepatic tissues has been directly implicated in excessive reactive oxygen species production and oxidative stress (26, 27) and in stimulating a range of fibrogenic action, including cell migration, cell hypertrophy and proliferation, secretion of proinflammatory cytokines, and extracellular matrix production [24, 26, 27, 53]. In both organs, the direct consequences of the AT_1_ receptor upregulation involves the over-stimulation of the profibrotic cytokine transforming growth factor-*β*, which seems to orchestrate the development and progression of diabetes-associated hepatic fibrosis and nephropathy [53].

A major observation of the present investigation was the significant downregulation of the AT_1_ receptor expressed in renal and hepatic tissues of green-tea-treated diabetic rats. Compared to the control diabetic rats, the expressed levels of the AT_1_ receptor in the green-tea-treated diabetic group were significantly reduced throughout renal cortical and medullary vascular and tubular segments, as well as hepatic lobules and portal tracts, to levels comparable to those observed in normal rats. Recently, the efficacy of green tea catechin treatments in attenuating diabetes-induced build-up of tissue-advanced glycation end products, and the subsequent generation of free radicals through autoxidation of glucose and glycated proteins has been reported [54]. As suggested by the present observations, the capacity of green tea in modulating diabetes-induced AT_1_ receptor upregulation may also imply beneficial interference in the deleterious effects of excessive AT_1_ receptor signaling, which is central in mediating excessive tissue remodeling and alterations in renal hemodynamic and tubular functions, manifested by the development of nephropathy and hepatic fibrosis [4–27, 52, 53]. In this regard, our observations may be supported by the documented therapeutic potential of AT_1_ receptor blockers not only in the management of progressive diabetic nephropathy [28] but also in alleviating diabetes-associated hepatic fibrosis [55]. Future studies on the significance of constituent green tea polyphenols may clarify the mechanisms that underlie their beneficial capacity in modulating the expression of AT_1_ receptor molecules implicated in diabetes-associated disorders.

## Figures and Tables

**Table 1:** Effect of green tea on kidney and liver catalase in diabetic rats.

Group (*n* = 8)	Mean ± SEM (units/mg Protein)	
	Kidney	Liver
Normal control rats	0.317 ± 0.023	0.929 ± 0.09
Diabetic control rats	0.162 ± 0.038^a^	0.493 ± 0.042^a^
Diabetic + green tea rats	0.252 ± 0.038^a,b^	0.575 ± 0.070^a,b^

^a^Significantly different from normal control rats.

^b^Significantly different from diabetic control rats.

**Table 2:** AT_1_ receptor quantification.

	Normal control rats	Diabetic control rats	Diabetic + green tea rats
Kidney			
Cortex	6,421 ± 541	10,825 ± 447*	5,101± 375
Outer medulla	6,567 ± 677	11,074 ± 305*	6,212 ± 715

Liver	2,514 ± 240	5,815 ± 635*	2,014 ± 670

Values are means ± SEM (*n* = 4). Each value represents the number of pixels that exceeded an arbitrary staining threshold per 300 × 300 pixel area. *P < 0.05 versus geen tea-treated diabetic rats and normal rats.
